# The role of ant nests in European ground squirrel’s (*Spermophilus citellus*) post-reintroduction adaptation in two Bulgarian mountains

**DOI:** 10.3897/BDJ.7.e38292

**Published:** 2019-10-07

**Authors:** Maria Kachamakova, Vera Antonova, Yordan Koshev

**Affiliations:** 1 Institute of Biodiversity and Ecosystem Research at Bulgarian Academy of Sciences, Sofia, Bulgaria Institute of Biodiversity and Ecosystem Research at Bulgarian Academy of Sciences Sofia Bulgaria

**Keywords:** anthills, *Lasius flavus*, mountain, reintroduction, *Spermophilus citellus*, symbiosis

## Abstract

The European ground squirrel (*Spermophilus citellus*) is a vulnerable species, whose populations are declining throughout its entire range in Central and South-Eastern Europe. To a great extent, its conservation depends on habitat restoration, maintenance and protection. In order to improve the conservation status of the species, reintroductions are increasingly applied. Therefore, researchers focus their attention on factors that facilitate these activities and contribute to their success. In addition to the well-known factors like grass height and exposition, others, related to the underground characteristics, are more difficult to evaluate. The presence of other digging species could help this evaluation. Here, we present two reintroduced ground squirrel colonies, where the vast majority of the burrows are located in the base of anthills, mainly of yellow meadow ant (*Lasius flavus*). This interspecies relationship offers numerous advantages for the ground squirrel and is mostly neutral for the ants. The benefits for the ground squirrel, including reduced energy demand for digging, as well as additional surveillance and hiding places available, could greatly enhance the post-reintroduction adaptation process.

## Introduction

The European ground squirrel or the souslik (*Spermophilus citellus*), is a rodent inhabiting pastures and meadows in Central and South-Eastern Europe. It constructs complex burrows up to 2 m deep (Brinkmann 1951, Ruzič 1978) where it hides, rests, reproduces and hibernates. During the last decades, the souslik's populations throughout its entire range are declining mainly because of habitat destruction and degradation (Coroiu et al. 2008). Currently it is listed as “Vulnerable” in the IUCN Red List and conservation measures have been taken all over its range. They often include conservation reintroductions – reestablishing the species in places where it was extinct. Most of the ground squirrel reintroductions in Bulgaria were organised by nature parks directorates and implemented in the mountains (Koshev et al. 2019).

Playing a key role in the ecosystem, the souslik lives in close interaction with several invertebrate species inhabiting its burrows - scarabid beetles (Carpaneto et al. 2011) and ectoparasites such as ticks (Honzakova et al. 1980) and fleas (Ryba et al. 1980). Some of these relationships are highly specific and not always reappear after reintroduction (Lindtner et al. 2019). Shabanova et al. (2014) also reported ants, as well as digger wasps and beetles, using sousliks’ burrows. The possible symbiosis with ants is especially interesting as they are also key ecosystem species, soil engineers and dominate in almost every terrestrial habitat. They may change the landscape by creating networks of soil macropores and building stationary, perennial mounds with altered physico-chemical structure and change the availability of resources for plants and animals (Alonso 2000, Ehrle et al. 2017). The nest’s structure (tunnels and chambers) allows water and gas circulation (Folgarait 1998). All these characteristics could be beneficial for the ground squirrels and possibly be linked to the conservation practice. Hapl et al. (2006) mentioned that the mounds of *Lasius flavus* could be used for initial hiding places in Slovakia for disorientated reintroduced sousliks, but the authors did not provide any references or further explanations.

Interactions between ants and small mammals, such as rodents and shrews, have rarеly been documented (Panteleeva et al. 2016, Gilev and Nakonechny 2010, Scherba 1965, Mironov 1986). Often they concern the competition between ants and rodents for seeds (e.g. Brown et al. 1979). Vygonyailova (2011) reports the superficial and surrounding mound’s material of red wood ants is attractive food for rodents, possibly because of the higher organic components.

Taking into account these scare data, our goal is to report a confirmed case of an interspecific relationship between souslik and ants and to discuss its implications in the conservation reintroduction practice.

## Materials and methods

Reintroduced colonies of ground squirrel in Bulgaria are regularly monitored. In 2017, it was observed that the majority of the holes were located in the base of anthills in two of these colonies (20.06.2017 in Bulgarka Nature Park and 30.06.2017 in Vrachanski Balkan Nature Park). The observations were confirmed in June 2018. On 11.06.2019 (Bulgarka Nature Park) and on 29.05.2019 (Vrachanski Balkan Nature Parka), the colonies were investigated in detail – multiple transects were designed in order to count and map the burrows and determine what percentage of them is associated with ant nests. The transects were chosen randomly, aiming to cover the entire area of the colonies (Suppl. materials 1, 2). When no more burrows were found, the transect was finished. Both holes of *S. citellus* and *Microtus* sp. were observed (identification based on holes' size and size and the surrounding excrement) but only the first type was considered in the current study. Each ant nest with a burrow was investigated - a small hole was made at the nest’s top and 3 to 5 ants were taken, fixed in 75% alcohol and identified under a microscope. The walls of the burrow at the entry hole were scraped, using a small garden shovel in order to check if the ants were present in the burrow’s interior. The behaviour of the ground squirrels was observed with binoculars for several minutes before the start of the transects.

Located on the northern slopes of Central Stara Planina mountain, Bulgarka Nature Park occupies 236.9 km^2^ with an average altitude of 870 m. Most of the park area (89%) is covered by deciduous forests and only the highest parts, close to the park's borders, are meadows. Ground squirrel colonies have been previously documented there (V. Popov – personal data for 2003, Koshev 2013). In 2013, the park’s directorate started a reintroduction programme in one such locality - Karamandra (42.7410N; 25.2510E, 1410 m a.s.l.). For the next three years, 149 animals were translocated there (Koshev et al. 2019).

The Vrachanski Balkan Nature Park is located in western Bulgaria, in the Stara Planina mountain. Its area is 288.03 km^2^ and the average altitude is 700 m (Bechev and Georgiev 2016). The species disappeared at the end of the 1950s due to a ban on transhumance (G. Stoyanov - unpubl. record). In 2013, park's authorities started a ground squirrel reintroduction project near Parshevitsa hut (43.1379 N; 23.4855 E, 1420 m a.s.l.) where there were historical data of its presence in the past. Until 2016, 132 individuals in total were translocated in order to form a new colony (Koshev et al. 2019).

The habitat in both locations is similar - mesophyte mountain meadows with diverse vegetation including both dicotyledonous and monocotyledonous plants. It is on a southern slope with a limestone base. A common species covering most of the ant nests is the *Thymus* sp. A moderate level of livestock grazing occurs (cattle in Bulgarka and horses in Vrachanski Balkan).

## Results

In **Bulgarka** Nature Park, 82.3% of all 132 burrows mapped were at the base of ant mounds (Fig. 1a, b). The most abundant ant species was *Lasius flavus* (65% active *L. flavus* nests of all nests with burrows) (Table 1). The rest of the nests with burrows were probably also built by the yellow meadow ant but currently abandoned - empty or occupied by other ant species. There was usually one (95%) and rarely two holes at one nest (Table 1). Nests of *Formica pratensis* were also observed, but there were no souslik burrows in the immediate vicinity. The area where the colony was detected in 2017 is 750 m distant from the release site and is densely covered with *Lasius flavus* nests. On the release site, only two ant nests were found.

In the **Vrachanski Balkan** colony, 84 active burrows were counted, 59 of them (70.2%) being in the bаse of anthills (Fig. 1c, d). In some nests, *Myrmica scabrinodis* or *Tetramorium* sp. were co-existing with the yellow meadow ant (Table 1). There were cases when abandoned *L. flavus*' nests were entirely occupied by other ant species. Burrows were also found near nests of the dominant protected ant species *Formica pratensis*, but not in it. The number of burrows were from one (97%) to three per nest (Table 1).

At both locations, the active nests of *Lasius flavus* were covered with vegetation, mainly *Thymus* sp. (in accordance with Bernard 1968). The mounds were with diameter between 30 cm and 150 cm (average 89 cm) and height between 8 cm and 50 cm (average 25 cm). Ants were not detected in the burrow’s interior after scraping. Surveillance behaviour of *S. citellus* (including juveniles) was observed at the top of the ant nests at each observation session done before the start of the transects.

## Discussion

As the reintroduced animals were taken from the plain - 480 m a.s.l. for Bulgarka and 100 m a.s.l. for Vrachanski Balkan (Koshev et al. 2019), the new habitat presented multiple difficulties for their adaptation. Artificial burrows (at least 5 per released individual) were prepared but the entire burrow system had to be re-established. In these conditions, each facilitating factor was crucial. The presence of the anthills should be considered as such a factor. The soil into and under the nest is more friable and drained and the vegetation on the nest is not so dense and tough. These factors greatly reduce the digging effort for initiating the tunnel. Not only the physical characteristics in and around the mound are specific, but also the chemical constituents (Bierbaß et al. 2015) - pH is frequently higher (King and Woodell 1975) and the microbiological - bacteria, fungi, actinomycetes and micro-arthropod communities differ (Sharma and Sumbali 2013). The last may have an indirect impact creating a cleaner micro-environment. We also know that the soil in the mounds, constructed by the ground squirrels themselves during excavation activities, has a higher pH and reduced microbiological acivity, diversity and heterogenity (Lindtner et al. 2019b). On the other hand, the formic acid, an organic substance of ants (subfamily Formicinae, including *Lasius* genus) mainly used for defence, trail marking and predation (Hölldobler and Wilson 1990), has repellent properties. Some authors reported about vertebrates which use formic acid for repellent (Falótico et al. 2007). The animals rub their fur or plumage with ants (so-called “anting” process) to avoid ticks (Acari) and other ectoparasites.

In addition, as part of the micro-relief of the habitat, the ant nests provide two additional benefits for the souslik. They are up to 50 cm tall and, when digging in their base, the animals are partly protected from predators, especially from raptors. Gedeon et al. 2012 showed that, in the new environment, the newly reintroduced sousliks prefer patches with higher grass cover that potentially also have a shelter role. On the other hand, the tops of the mounds of *L. flavus* are thick, covered with vegetation and there are no ants on the surface. In consequence, the *S. citellus* use them as watchtowers. In both locations, surveillance behaviour was observed on the top of the ant nests during the observation session. Such features are especially important during the summer when, despite livestock grazing, the grass could become as tall as 50 - 60 cm and the visibility for the ground squirrels drops. Considering all these positive effects, we could at least partly explain the settling of the Bulgarka colony 750 m distant from the release site with searched cohabitation with ants. The average nests’ dimensions showed that they are more than 10 years old - the nest’s diameter increases by 7 cm/year and the height by 2.2 cm/year (Waloff and Blackith 1962). Therefore, they were present well before the reintroduction activities started. This was also confirmed by the park’s authorities.

These multiple benefits are not associated with conflict with the ants. Potential conflict seems probable with the territorial ants *Formica pratensis*, but not with the peaceful *L. flavus*. Scherba (1965) reports a case when the ants do not avoid and do not attack the voles and both live in a “myrmecocole” interaction. The author considers the microhabitat formed in ants nests is used by the voles only when the most suitable habitats are already occupied. Trophic competition may arise with the red wood ants for small invertebrates (snails, beetles, worms etc.) and with the harvester ants for seeds (O'Dowd and Hay 1980, Brown et al. 1979, Abramsky 1983, Davidson et al. 1984). According to Panteleeva et al. (2016), the red wood ants may disturb the digging activity of rodents and may compete for small invertebrates. The case with yellow meadow ant is different. *L. flavus* is feeding underground with aphids, excretions of root-living aphids and coccids (King and Woodell 1975). Their nests are about 15 - 20 cm deep (Steinmeyer et al. 2012) so the souslik tunnel system is developed below it. As there were no ants present even in the upper part of the tunnel, we suggest that the ants’ structures, destroyed during the initial digging, are quickly sealed and the direct interactions between the two species are limited. There are scarce data on the presence of ants in the *S. citellus*' diet - Formicidae species (*Tetramorium caespidum, Lasius alienus, Tapinoma erraticum, Solenopsis fugax, div. Puppen, Myrmica scabrinodis, M. rugulosa)* have been identified in gut contents and droppings, but their share is not considerable - 11% of all insects found in the diet (Herzig-Straschil 1976). Nevertheless, we did not find any trace of scratching and digging on the nest tops - the vegetation was intact and the nests continue to be viable. It is possible that the ants have even some indirect benefits from the ground squirrel presence, related to general improvement of the ecosystem functioning and diversity (Dundas et al. 2018).

Based on the observations described, we would conclude that the relationship between the two species (*Spermphilus citellus* and *Lasius flavus*) could be considered as commensalism (see Dickman 1992). We suggest that the souslik has numerous benefits from cohabitation with the ants and, for the ants, the interaction is neutral. Panteleeva et al. (2016) assumed that there is “coadaptation” (symbiosis, commensalism type) between the ants and the small rodents, that were using the same microhabitats. In the investigated case, it is also possible that the interactions have been enforced by the reintroduction event, as we have not observed it in the natural colonies until now.

With regard to the conservation status of the *S. citellus*, the mentioned benefits could have a role in helping the conservation efforts. Hapl et al. (2006) describe a hard-release method when additional cover of branches was put around the *Lasuis flavus*’ nests so that the sousliks could easily and safely dig their first holes after translocation. Nevertheless, nothing is reported about the scientific reasons on which this method is based, neither about the percentage of successful results. Therefore, our study provides a robust proof for the suitability of this practice. In addition, according to our observations, the yellow meadow ant and the souslik have common microhabitat preferences - in the investigated cases, the areas with anthills coincided with the souslik colony. The ant nests of *Lasius flavus* are also long-lasting structures - they can be active for between 22 and 150 years (Dostal 2005, Steinmeyer et al. 2012) and are densely situated - up to 2500 nests/ha (Elmes 1991). This means that the presence of *Lasuis flavus* mounds could serve as additional confirmation for the habitat suitability for *S. citellus* reintroduction. In conclusion, we state that the reported ecological findings could be beneficial for planning and implementation of future ground squirrel reintroduction projects.

## Supplementary Material

BED29E2D-A385-5AB4-9C5D-B0F085246A47 10.3897/BDJ.7.e38292.suppl1 Supplementary material 1 Transect and burrows in Vrachanski Balkan Nature Park Data type: map
File: oo_338199.pdf https://binary.pensoft.net/file/338199 Maria kachamakova

668AB5C9-827C-5603-BD93-A39EB1C0E11B 10.3897/BDJ.7.e38292.suppl2 Supplementary material 2 Transect and burrows in Bulgarka Nature park Data type: map
File: oo_338202.pdf https://binary.pensoft.net/file/338202 Maria Kachamakova

## Figures and Tables

**Figure 1a.**
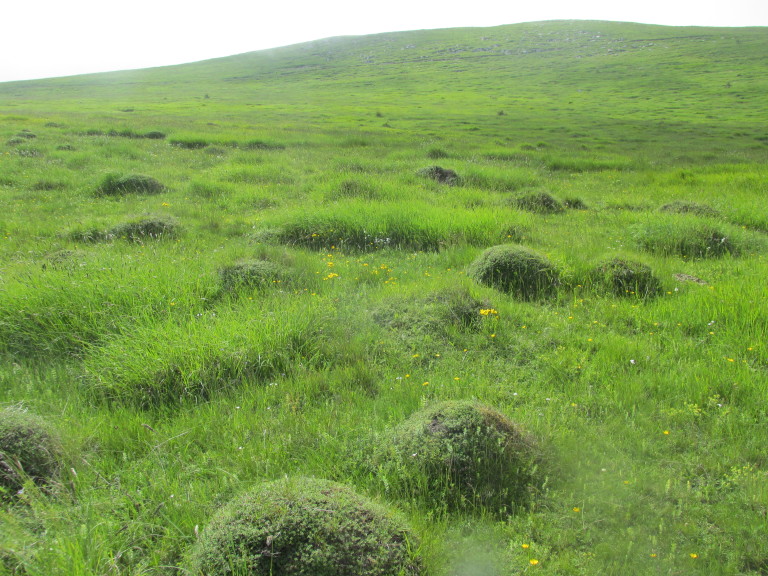
Nests of *L. flavus* in the colony in Bulgarka Nature Park

**Figure 1b.**
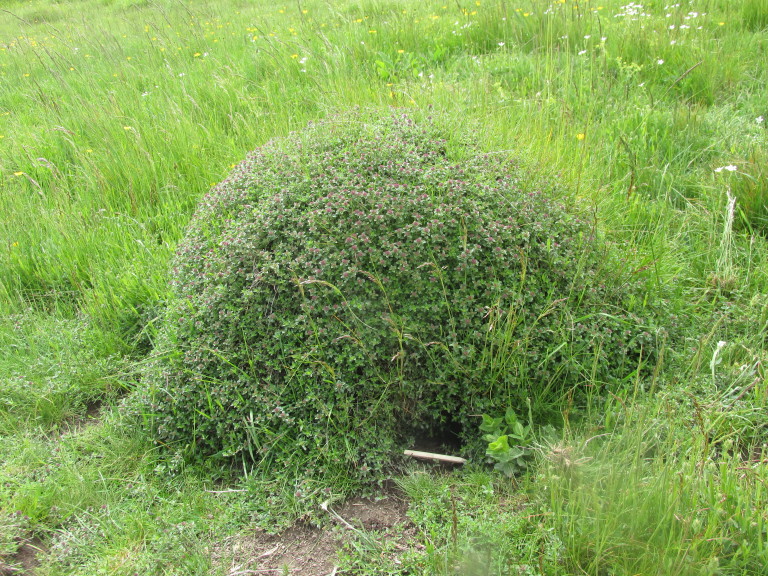
Nest of *L. flavus* with burrow in the base in Bulgarka Nature Park

**Figure 1c.**
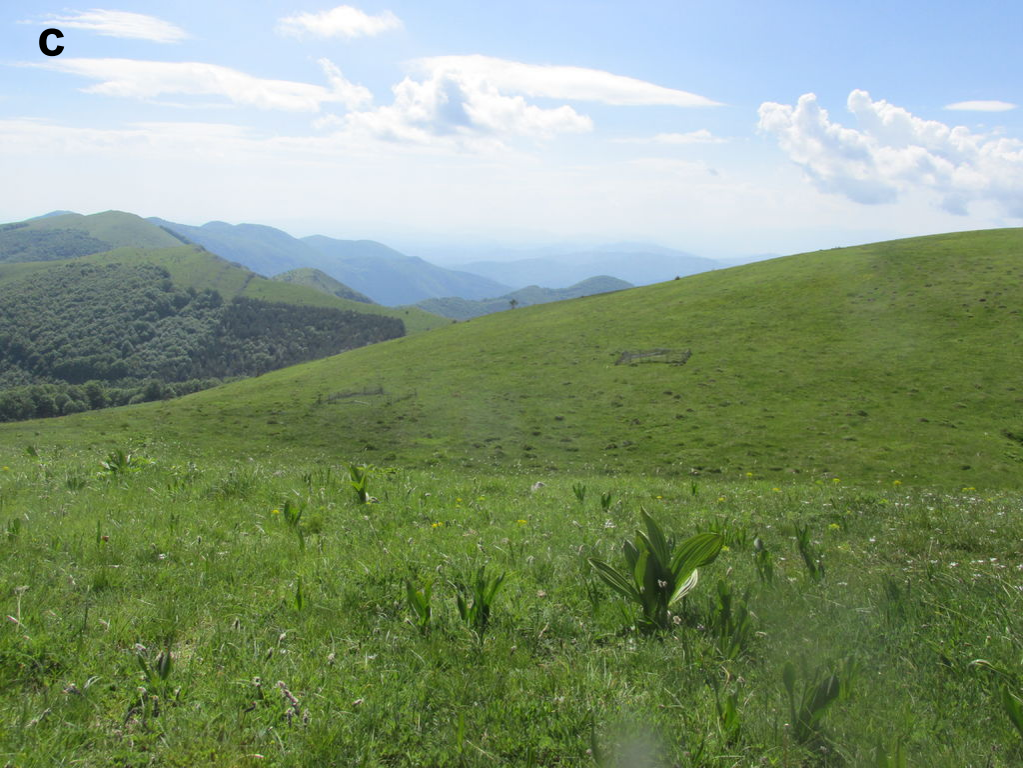
The reintroduced colony in Vrachanski Balkan Nature Park - a general view

**Figure 1d.**
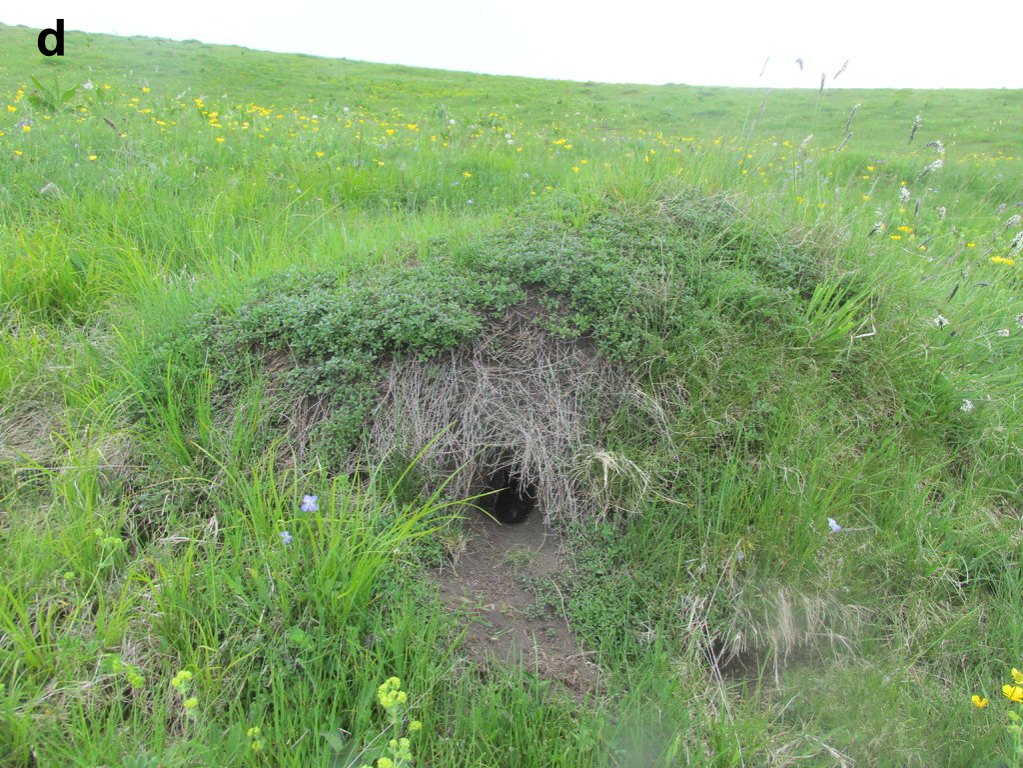
Nest of *L. flavus* with burrow in Vrachanski Balkan Nature Park

**Table 1.** Types of ant nests with burrows mapped in 2019.

	**Bulgarka Nature Park**	**Vrachanski Balkan Nature Park**
**Total number of burrows mapped**	**132**	**84**
**Transect length**	**5043 m**	**4398 m**
**Percentage of burrows in ant nests by ant species**:		
*Lasius flavus* nests	64.7%	57.1%
*Formica cunicularia* nests	-	1.2%
*Formica rufibarbis* nests	-	1.2%
Abandoned or very old nests (more than 10 years) of *L. flavus* occupied by *Lasius alienus*	-	6%
Abandoned or very old nests (more than 10 years) of *L. flavus* occupied by *Formica fusca*	-	3.5%
Abandoned or very old nests (more than 10 years) of *L. flavus* occupied by *Myrmica scabrinodis*	2.9%	-
Abandoned or very old nests (more than 10 years) of *L. flavus* occupied by *Tetramorium* sp.	2.9%	-
*Nests of Lasius flavus* where it was found coexisting with *Myrmica scabrinodis*	1.5%	-
*Nests of Lasius flavus* where it was found coexisting *with Tetramorium* sp.	1.5%	-
Empty nests	8.8%	1.2%
**Total percentage of burrows in ant nests**	**82.3**%	**70.2**%
Ant nests with one hole	95%	97%
Ant nests with two or three holes	5%	3%
