# Supplementary figures and images for: The role of ant nests in European ground squirrel’s (Spermophilus citellus) post-reintroduction adaptation in two Bulgarian mountains
Source: Biodivers Data J. 2019 Oct 7;7:e38292. doi: 10.3897/BDJ.7.e38292 (PMC6791899; doi:10.3897/BDJ.7.e38292)

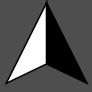

### Legend

- 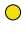 Burrows
- 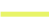 Transect

### Altitude

- 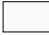 50
- 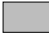 500
- 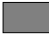 1000
- 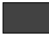 1500
- 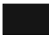 2000

25 0 25 50 75 100 m

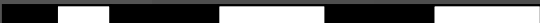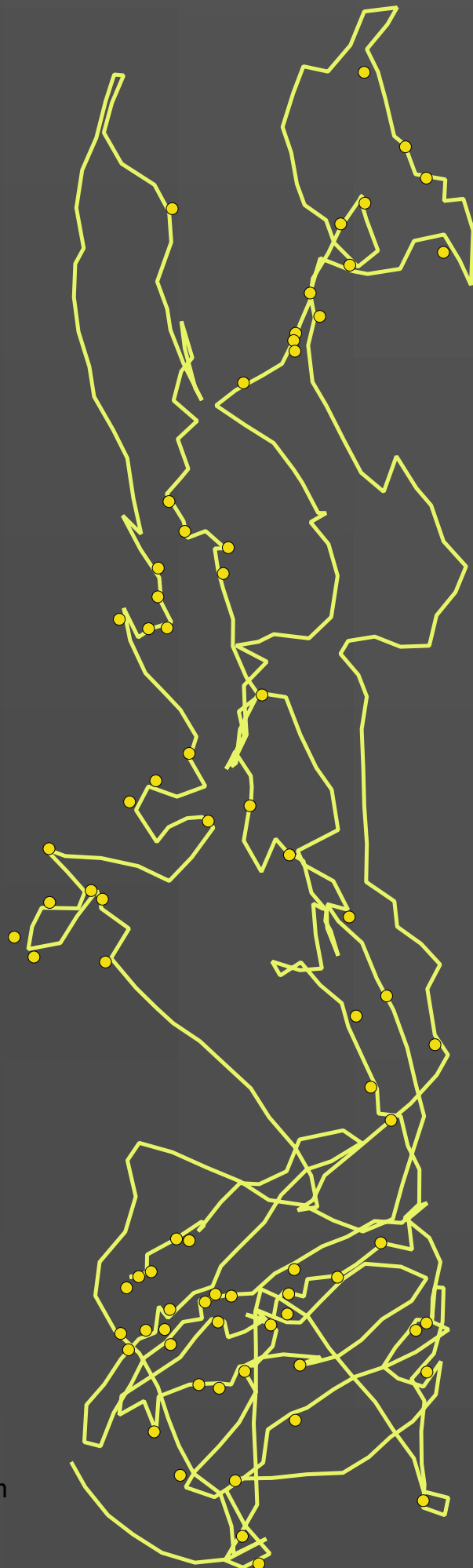

Supplement: Supplementary material 1 — Transect and burrows in Vrachanski Balkan Nature Park [file bdj-07-e38292-s001.pdf]

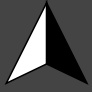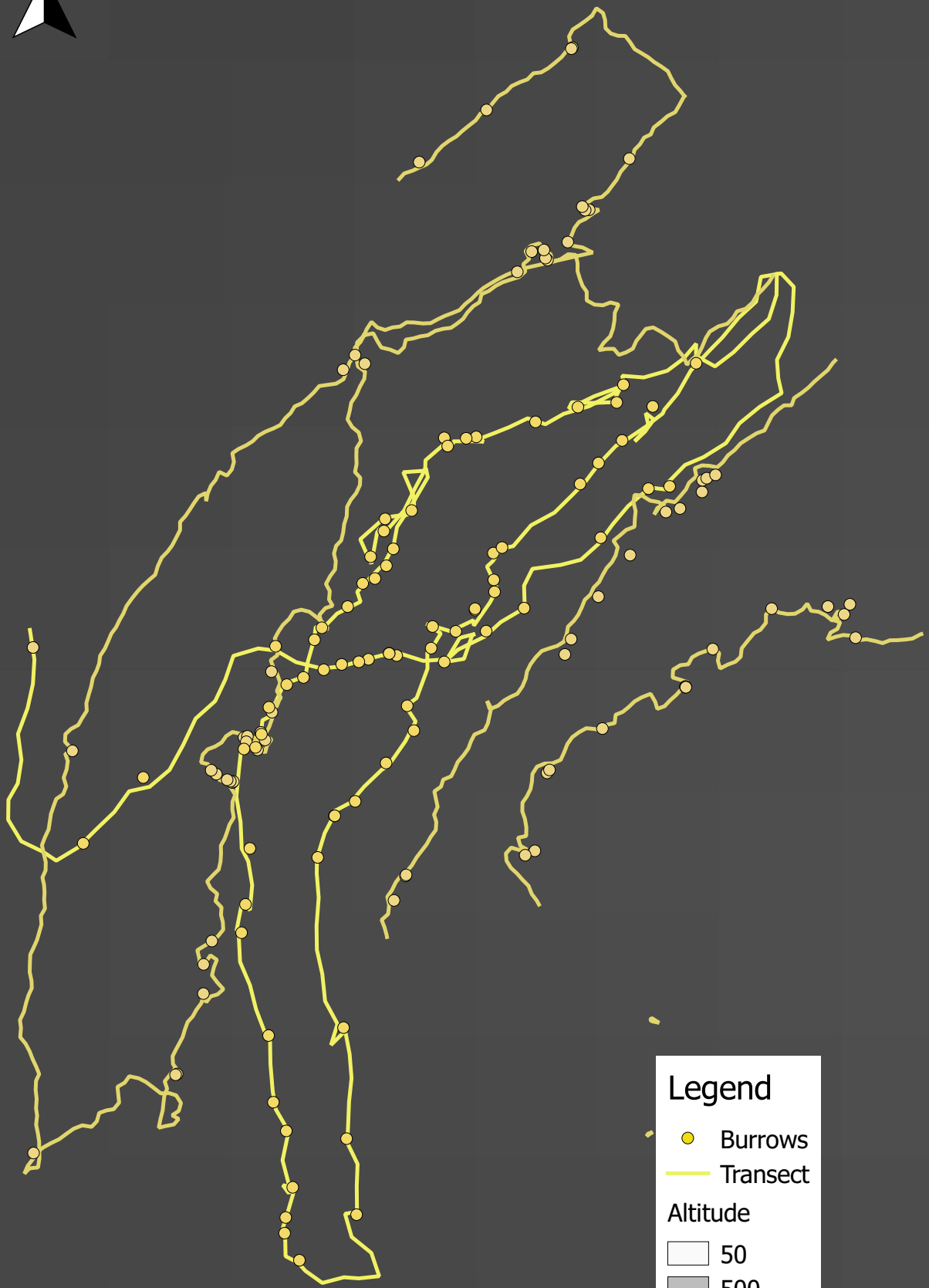

Legend

● Burrows

— Transect

Altitude

50

500

1000

1500

2000

25 0 25 50 75 100 m

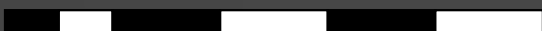

Supplement: Supplementary material 2 — Transect and burrows in Bulgarka Nature park [file bdj-07-e38292-s002.pdf]
